# Competition between Superconductor – Ferromagnetic stray magnetic fields in YBa_2_Cu_3_O_7−x_ films pierced with Co nano-rods

**DOI:** 10.1038/s41598-017-05909-6

**Published:** 2017-07-18

**Authors:** V. Rouco, R. Córdoba, J. M. De Teresa, L. A. Rodríguez, C. Navau, N. Del-Valle, G. Via, A. Sánchez, C. Monton, F. Kronast, X. Obradors, T. Puig, A. Palau

**Affiliations:** 1 Institut de Ciència de Materials de Barcelona, CSIC, Campus de la UAB, 08193 Bellaterra, Spain; 2 Instituto de Ciencia de Materiales de Aragón (ICMA), Universidad de Zaragoza-CSIC, E-50009 Zaragoza, Spain; 3 Laboratorio de Microscopías Avanzadas (LMA), Instituto de Nanociencia de Aragón (INA), Universidad de Zaragoza, 50018 Zaragoza, Spain; 4 Department of Physics, Universidad del Valle, A.A. 25360 Cali, Colombia; 5 Department Física, Universitat Autónoma de Barcelona, 08193 Bellaterra, Spain; 6 Department of Physics and Astronomy, University of Texas at San Antonio, San Antonio, TX 78249 USA; 7 Helmholtz-Zentrum Berlin für Materialien und Energie, Albert-Einstein-Strasse 15, D-12489 Berlin, Germany; 8 CEMES-CNRS 29, rue Jeanne Marvig, B.P. 94347, F-31055 Toulouse Cedex, France; 9 Institute of Mathematics and Statistics, University of Sao Paulo, Butantã, Brazil

## Abstract

Superconductivity and ferromagnetism are two antagonistic phenomena that combined can lead to a rich phenomenology of interactions, resulting in novel physical properties and unique functionalities. Here we propose an original hybrid system formed by a high-temperature superconducting film, patterned with antidots, and with ferromagnetic nano-rods grown inside them. This particular structure exhibits the synergic influence of superconductor (SC) - ferromagnetic (FM) stray fields, in both the superconducting behaviour of the film and the three-dimensional (3D) magnetic structure of nano-rods. We show that FM stray fields directly influence the critical current density of the superconducting film. Additional functionalities appear due to the interaction of SC stray fields, associated to supercurrent loops, with the non-trivial 3D remanent magnetic structure of FM nano-rods. This work unravels the importance of addressing quantitatively the effect of stray magnetic fields from both, the superconductor and the ferromagnet in hybrid magnetic nano-devices based on high temperature superconductors.

## Introduction

Extensive efforts have been devoted over the past years to explore the interaction of superconductivity and ferromagnetism in hybrid systems, especially at the nanoscale. Unique behaviours have been found by combining these antagonistic phenomena, offering the possibility to obtain new solid state devices^1–3^. The coexistence of these two phases in uniform materials requires singular conditions and occurs only in a limited number of systems^4–7^. Multifunctional hybrid materials have been widely explored as an efficient route to design materials, offering an ideal approach to study Superconductor (SC) – Ferromagnetic (FM) interplay. Most of the work performed up to now has focused towards understanding coupling of SC-FM in hybrid systems with conventional low temperature superconductors. In addition to interactions with electronic level and proximity effects^8–12^, the stray fields originated from FM materials may directly influence the superconducting properties^13–18^. Some of these microscopic mechanisms can be used to modify pinning forces in the SC material and enhance their critical current performance^19–23^ or tune the pinning potentials to control and manipulate vortex dynamics^24–28^. For high-temperature superconductors (HTS), much attention has been devoted exploring the possibility to enhance intrinsic flux pinning from the spatial modulation of the local magnetic field in SC-FM multilayers^29–31^ or by incorporating FM inclusions within the SC matrix^32^. Although hysteretic effects have been observed in hybrid HTS structures induced by SC-FM interactions^33–35^, the present understanding of those systems is very limited. In particular, the self-interaction between the FM magnetization and the magnetic field associated to supercurrents is almost unexplored. In this direction, a novel strategy to generate, modify, and annihilate a large number of singular spin configurations was recently proposed, by combining HTS patterned films with a soft magnetic thin film grown on top^36^. Control and motion of nanoscale magnetic patterns and domain walls are a hot research topic for its considerable potential in next-generation spintronic devices nowadays^37–40^.

The aim of this work is to establish an experimental system in which SC-FM mutual interactions can be studied in a model HTS hybrid system formed by an inhomogeneous superconducting film combined with three-dimensional (3D) ferromagnetic nano-rods. The system designed consists of a YBa_2_Cu_3_O_7−x_ (YBCO) film patterned with antidots and filled with ferromagnetic Cobalt (Co) nano-rods piercing the superconducting film. On the one hand, YBCO films are technologically one of the most relevant HTS, possessing many intrinsic defects able to trap high magnetic fields^41^. On the other hand, Co nano-rods grown by focused electron-beam induced deposition (FEBID), offer the possibility to generate 3D spin textures that could provide a third dimension in controlling magnetic states at the nanoscale^42, 43^. We have studied mutual interactions coming from both FM nanostructures that induce local inhomogeneous stray fields within the SC matrix, *H* ^*FM*^, and trapped stray magnetic fields generated by SC current loops, *H* ^*SC*^. This unique system, based on an in-homogeneous HTS thin film, with high intrinsic pinning and patterned with antidots, will induce largely trapped fields, that will be of the order of FM stray fields. Competition between these two magnetic fields give place to explore novel SC-FM interactions which do not appear in other studied systems based on conventional low temperature superconductors, were trapped SC magnetic fields are essentially negligible. We have combined electric transport measurements with transmission X-ray photoemission electron microscopy (T-XPEEM) to elucidate the interplay between competing superconducting and ferromagnetic stray fields. Experimental results are supported by micromagnetic simulations which illustrate the unique potential of FEBID to grow nano-rods with complex 3D remanent magnetization states, that may be modified with SC stray fields.

## Results and Discussion

**Figure 1:**
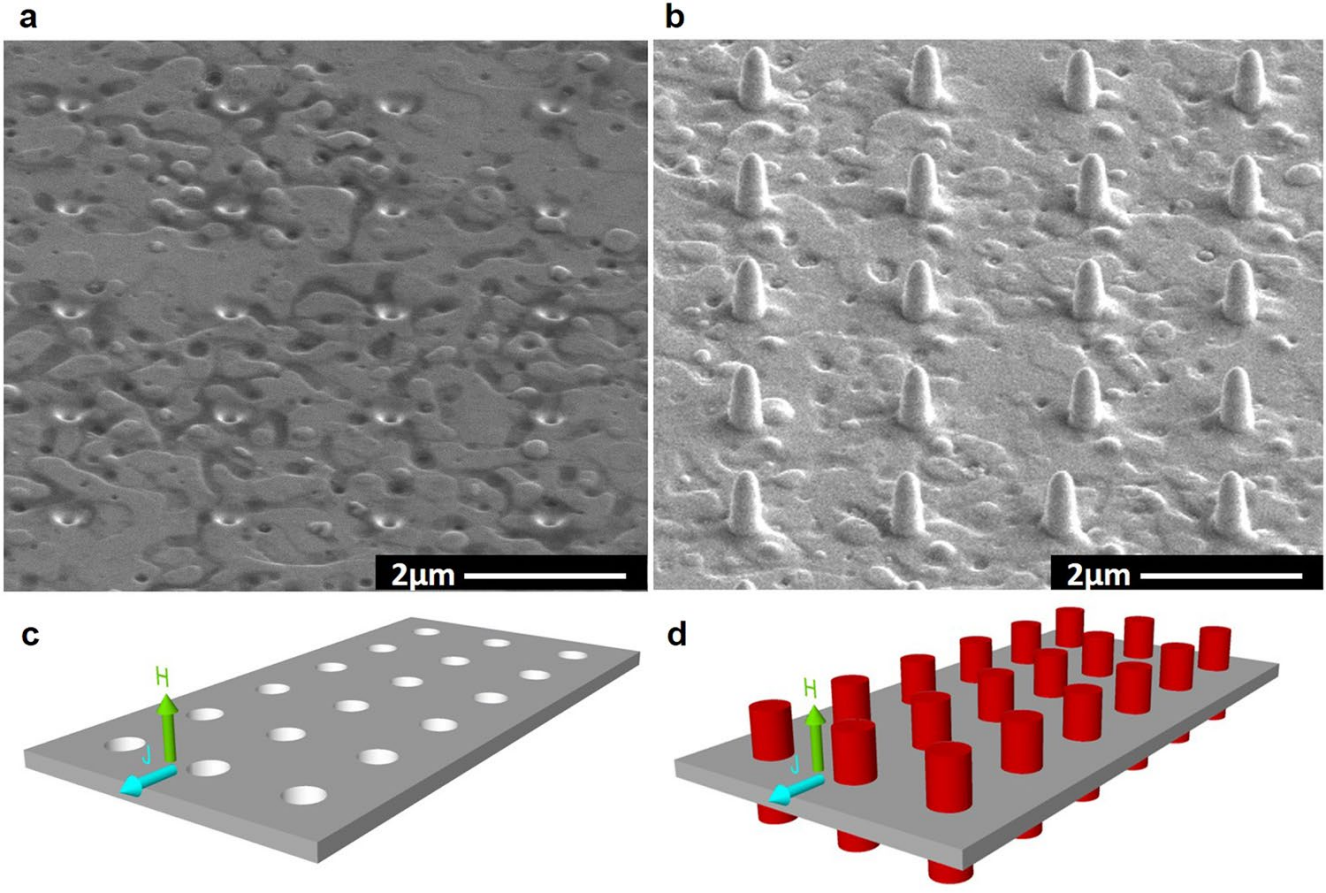
Sample description. Tilted SEM pictures of YBCO transport bridges patterned with an array of (**a**) antidots (**b**) antidots filled with Co nano-rods. (**c**) and (**d**) Schematic representation (not at scale) of transport bridges shown in (**a**) and (**b**), respectively, with the direction of applied electric current and magnetic field indicated.

The studies have been performed in a 125 nm YBCO thin film, patterned with three equal transport bridges patterned in a 4-point geometry. We used one bridge as a reference while in the other two we milled an array of circular antidots. These antidots of 200 nm in diameter and about 425 nm in depth (completely perforating the film and part of the substrate) were distributed in a square lattice with inter-dot distance 2 μm (Fig. 1a). In one of the two bridges, the antidot holes where filled with Co nano-rods grown by FEBID, overhanging about 300 nm from both sides of the YBCO layer (Fig. 1b). From now on we will refer to the superconducting system patterned with antidots as SC^A^ and the hybrid system with antidots filled with Co rods as SC^A^-FM^R^. A schematic representation of the two YBCO bridges with the applied current and magnetic field directions indicated is shown in Fig. 1c,d. The self-field critical current density of the reference bridge at 65 K, *J*_*c*_^*sf*^ = 8.4 MA/cm^2^ was reduced to 3 MA/cm^2^ and 1.5 MA/cm^2^ in SC^A^ and SC^A^-FM^R^, respectively. The obtained *J*_*c*_^*sf*^ values, calculated from the full bridge cross section, are lower than the expected ones, according the reduction in the effective cross sectional area due to the presence of antidots. The value of *T*_*c*_ is much less affected with a decrease of less than 5 K observed in both cases, indicating that the *J*_*c*_^*sf*^ reduction is mainly associated to a local amorphization of the material in the area surrounding the antidots and nano-rods.

### Hysteretic Critical current densities

**Figure 2:**
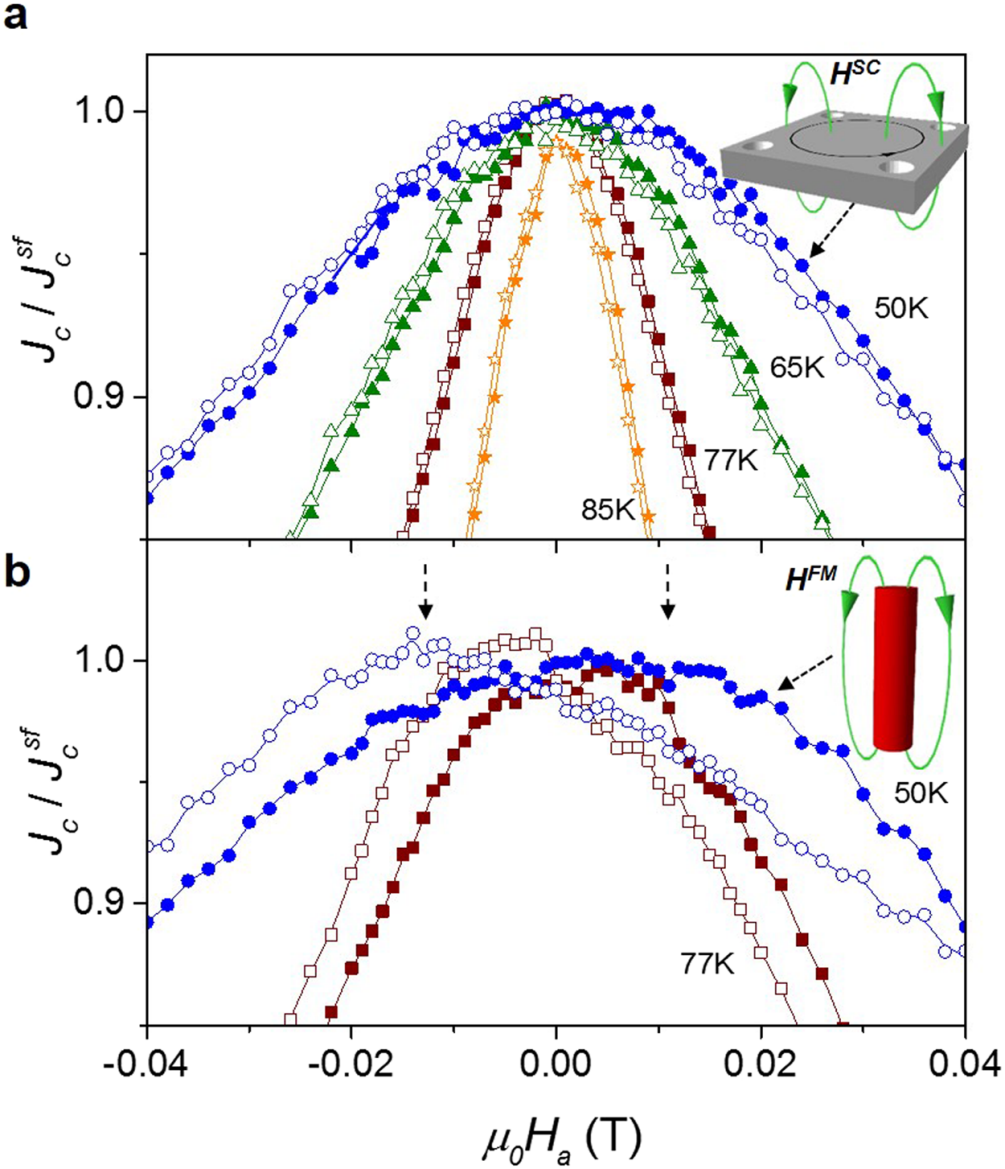
Hysteretic behaviour of J_c_. Normalized critical current density vs. applied magnetic field at different temperatures for a bridge with (**a**) antidots and (**b**) antidots filled with nano-rods, measured by decreasing field (closed symbols) and increasing field (open symbols). Dashed vertical arrows indicate the value of *H* ^*peak*^ for the *J*_*c*_ *(H*_*a*_ *)* curves measured at 50 K. Upper and lower insets show a schematic representation of trapped stray fields generated by SC loops, *H* ^*SC*^, and Co nano-rods, *H* ^*FM*^, respectively, in the first quadrant of closed symbols.

As an aid to understanding the effect of the antidot patterning and the presence of nano-rods in the magnetic field distribution within the YBCO film, we have evaluated the critical current density dependence with magnetic sweep direction. Figure 2 shows the transport critical current density, *J*_*c*_, normalized to its self-field value, as a function of the applied magnetic field, *H*_*a*_, at different temperatures for SC^A^ and SC^A^-FM^R^. In both systems, the *J*_*c*_(*H*_*a*_) curves were measured by applying a maximum field + *H*_*a,max*_ in a zero-field cooling process and sweeping it from + *µ*_*0*_ *H*_*a,max = *_0.1 T down to - *µ*_*0*_ *H*_*a,max*_ = − 0.1 T (closed symbols) and back to + *µ*_*0*_ *H*_*a,max*_ (open symbols). We observe that an appreciable hysteresis in *J*_*c*_(*H*_*a*_) is obtained for the sample with antidots, SC^A^, (Fig. 2a) that is clearly enhanced in the hybrid SC^A^-FM^R^ system, due to the presence of FM antidots (Fig. 2b).

**Figure 3:**
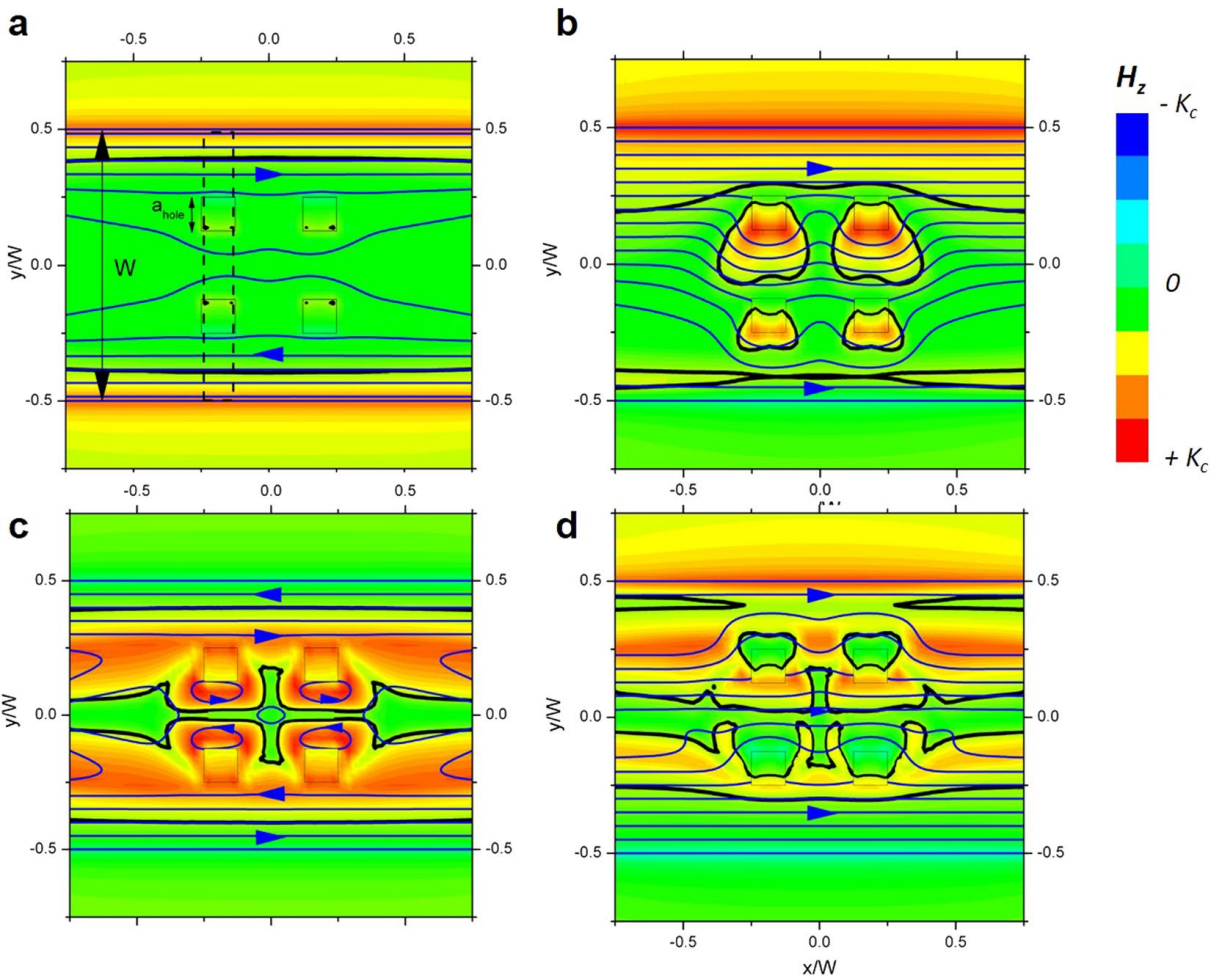
Inhomogeneous SC magnetic field distribution. Out-of-plane magnetic field *H*_*z*_ (colour scale) and current stream lines (blue lines, arrows indicate direction of current) induced in a thin straight superconducting film, of uniform thickness *t*, width *W* and constant critical current *J*_*c*_, with four symmetrical square antidots. For a uniform applied perpendicular positive magnetic field *H*_*a*_ = 0.3*K*_*c*_ (being *K*_*c*_ = *J*_*c*_ *t*) (**a**) after zero field cooled and (**c**) after applying a positive field to saturate the sample and then decreasing down to *H*_*a*_. (**b**) and (**d**) applying a transport current *I* = 0.7*K*_*c*_ *W* in situation reached in (a) and (**c**), respectively. Thick black lines separate the regions with *H*_*z*_ < *H*_*a*_ from *H*_*z*_ > *H*_*a*_.

In the case of SC^A^, the hysteretic behaviour in *J*_*c*_ can be attributed to an inhomogeneous internal magnetic field distribution within the SC bridge, induced by the presence of antidots. Numerical simulations have been used to evaluate the field in-homogeneities appearing when the film is subjected to different magnetic field sweeps. Our experimental system has been emulated by considering an infinite strip of width, *W*, with an array of 4 squared antidots of size, *a*_*hole*_ = *W*/8, simulating the two sequences. In the first one, a perpendicular magnetic field, *H*_*a*_ *<* *J*_*c*_ *t*, is applied to the sample in the virgin state (Fig. 3a), followed by the application of a longitudinal transport current (Fig. 3b). In the second sequence, the applied field is first raised up to a high value to saturate the sample and then decreased back to *H*_*a*_ (Fig. 3c). Also in this case the same transport current is applied (Fig. 3d). When increasing *H*_*a*_ from the virgin state (Fig. 3a) we observe critical current to penetrate from all of the strip edges. These currents flow in the positive direction at the strip upper half and in the negative direction at its lower half. If the field is further increased and then reversed back to *H*_*a*_, new currents with opposite direction penetrate from the outer edges and SC current loops are generated in the regions between the antidots (Fig. 3c) producing large stray magnetic fields in these regions. The effect of applying a transport current to the sample in sequences (a) and (c) originates opposite bending of the current streamlines, producing very different field distributions at the first dissipation region (region between antidots indicated with dashed lines in Fig. 3a). If in the approximated case of constant *J*_*c*_ a significantly different current distribution (so different field distribution) is observed for the two sequences, we can assume that in the case *J*_*c*_(*H*) dependence the obtained current profile will also be different. Therefore, we predict that a hysteretic behaviour in the *J*_*c*_(*H*_*a*_) curve will be found for thin film samples with such geometry, as it is experimentally observed. No hysteresis was obtained in the reference bridge, within the experimental resolution (see Supplementary Fig. S1).

We next discuss the hysteresis obtained in the hybrid SC^A^-FM^R^ system in which two different internal field contributions have to be considered; the one associated to the stray fields trapped inside the patterned superconductor, described above for the SC^A^ system, added to the magnetic field contribution coming from the ferromagnetic nano-rods. According to this, a maximum in *J*_*c*_(*H*_*a*_) will occur at the applied magnetic field, *H* ^*peak*^, that locally compensates both, the average trapped stray field generated by SC current loops, < *H* ^*SC*^ > , and the average stray field coming from Co nano-rods, < *H* ^*FM*^ > ; i.e, *H* ^*peak*^ ∼ < *H* ^*SC*^ >  +  < *H* ^*FM*^ > . Insets in Fig. 2 show a schematic representation of the < *H* ^*SC*^ > and < *H* ^*FM*^ > stray fields, induced at the positive *J*_*c*_(*H*_*a*_) branch, after saturating the sample with 0.1 T and sweeping the magnetic field back to zero. The contribution of the superconductor will maintain this sign in the whole *J*_*c*_(*H*_*a*_) curve measured with a negative sweep (closed symbols), whereas the magnetization coming from the rods will change its sign below their coercive field. However, the qualitative effect of both stray fields in the J_c_ hysteresis, associated to the SC and magnetic hysteresis loops, will be the same.

**Figure 4:**
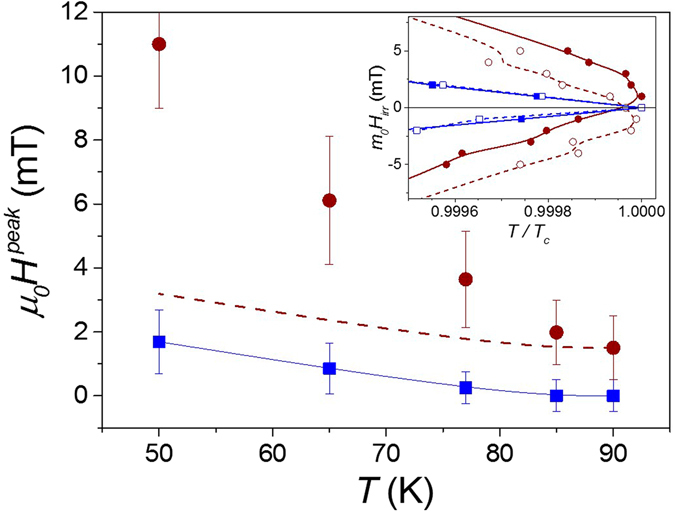
Temperature dependence of μ_0_H^peak^. Magnetic field position of the peak in the critical current density and critical temperature at different temperatures for a bridge with antidots (squares) and antidots filled with nano-rods (circles). Dashed line shows the temperature dependence obtained by sum of squared points and the contribution of nano-rods at high temperature. Inset shows the irreversibility line measured decreasing field (closed symbols) and increasing field (open symbols), for the bridge with antidots (squares) and antidots filled with nano-rods (circles).

Figure 4 shows the temperature evolution of *H* ^*peak*^ measured for both systems. The points at 90 K have been obtained by measuring the maximum irreversibility field. The inset in Fig. 4 depicts the irreversibility line (IL) obtained for SC^A^ and SC^A^-FM^R^ by sweeping the external field from a maximum magnetization value of + *µ*_*0*_ *H*_*a,max   *_=+0.1 T to −0.1 T and back to + 0.1 T. A conventional non hysteretic IL, with maximum *T*_*c*_ at zero field, is obtained for SC^A^, whereas a clear hysteresis is found for the hybrid SC^A^-FM^R^ system. Trapped magnetic fields generated by SC current loops in SC^A^, <*H* ^*SC*^>, (squares in Fig. 4) are proportional to *J*_*c*_ and thus follow a *J*_*c*_(*T*) dependence. No hysteresis is found at high temperatures (*T* > 80 K) while a substantial contribution of <*H* ^*SC*^ > ~2mT is obtained at 50 K. This trapped field behaviour appears similar to that measured in polycrystalline YBCO wires^44^ granular coated conductors^45, 46^ and artificial multi-granular YBCO films^47^, mediated by trapped flux retained by strong active pinning regions in non-homogeneous samples.

For the hybrid SC^A^-FM^R^ system, we need to consider the additional contribution associated with Co nano-rods stray field, < *H* ^*FM*^ > , which, according to the large curie temperature of the material (*T*_*c*_ = 1400 K for bulk Co), should be constant in the explored temperature range (from 50 K to 90 K, were *T* < *T*_*c*_/15), if no contribution of the superconductor is considered. Supplementary Fig. S2, show the dependence of *H* ^*peak*^, determined from the maximum of the irreversibility line as a function of the maximum applied field, with no influence of *H* ^*SC*^, were a saturation of *H* ^*FM*^ is observed above 100 mT. Recent M vs. H measurements performed in a Co nanorod of 80 nm of diameter grown by FEBID on top of a nano-squid^48^ showed temperature independent hysteresis loops (measured from 1 K to 60 K), which were saturated at 70 mT with a coercive field of ∼60 mT. Previous results obtained in 2D Co nanowires grown by FEBID showed a decrease of the coercive field with the wire diameter^49^, in agreement with the simulated hysteresis loop shown in Supplementary Figure S2. Thus, a temperature independent contribution of < *H* ^*FM*^ > , without influence of the superconducting film, would imply a continuous upward shift of *H* ^*peak*^ vs. temperature by comparing the two systems (dashed line in Fig. 4). This line has been determined from the value of H^peak^ evaluated from the shift in the irreversibility line (90 K point). However, a clear deviation from the expected behaviour is obtained, indicating a mutual interaction between SC and FM fields. In the field range where the peak is observed (2–12 mT) the nano-rod magnetization is well below saturation, with a considerable susceptibly (see Supplementary Figure S2). It is, thus, very plausible that the influence of the superconducting stray field, which is strongly temperature dependent, may modify the magnetic state of nano-rods, thus changing < *H* ^*FM*^ > . We observe that the contribution from < *H* ^*FM*^ > is strongly enhanced when SC current loops, and the associated stray fields < *H* ^*SC*^ > , appear to be important in the film (at low temperatures). These results reveal the mutual influence between the superconducting film and the nano-rods. The stray fields generated by SC current loops contribute to change the magnetization of Co nano-rods, increasing their associated stray fields, which at the same time determine the superconducting critical current density behaviour of the system.

### Remanent magnetic structure of nano-rods

**Figure 5:**
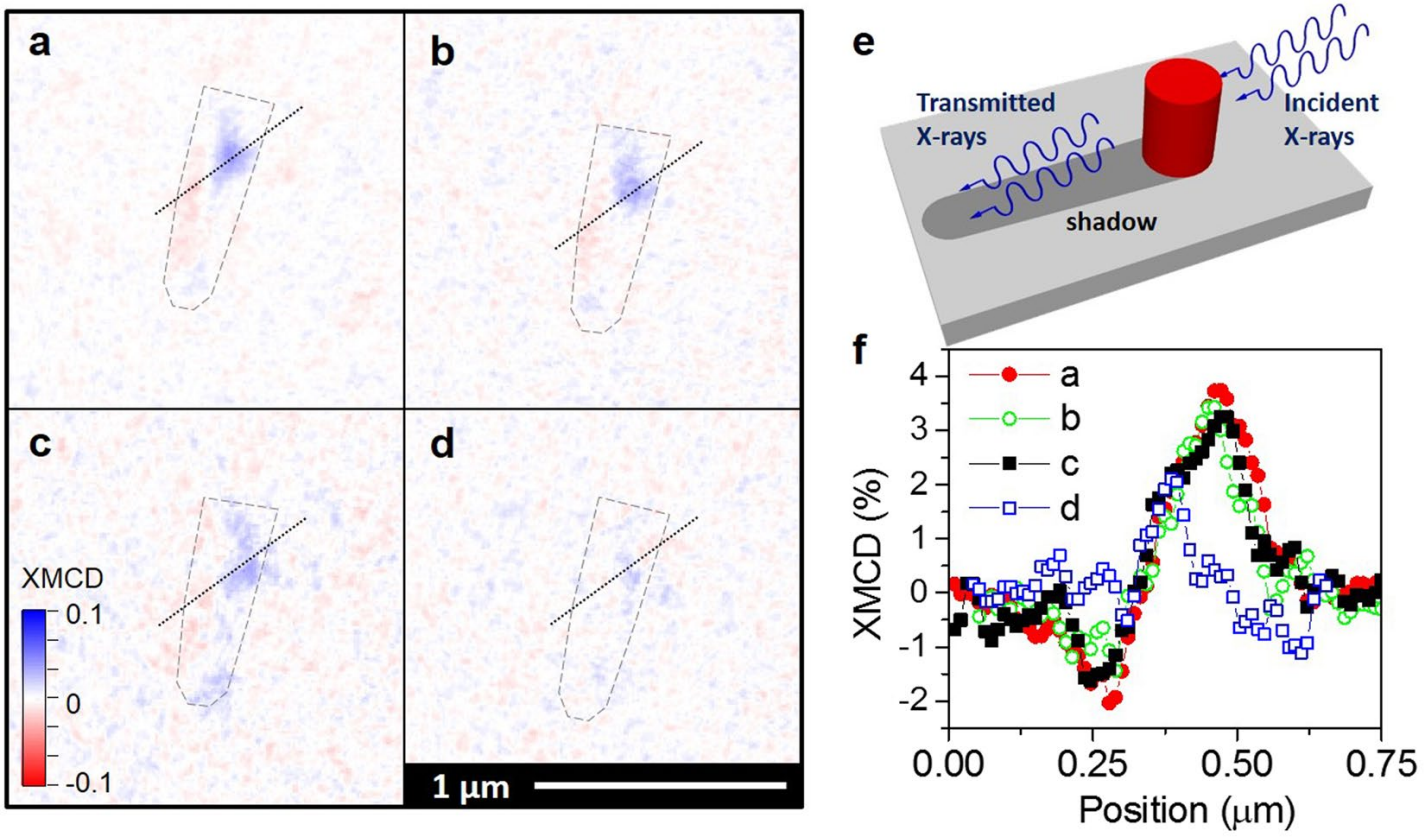
XMCD magnetic images. Magnetic structure obtained in remanence for a single Co nano-rod (**a**) at 300 K after a maximum applied field of −100 mT, (**b**) at 300 K, −50 mT, (**c**) at 65 K, 50 mT-ZFC, i.e. removing the magnetic field T > T_c_, and (**d**) at 65 K, 50mT-FC, applying and removing the magnetic field at 65 K. The colour bar indicates the direction of the magnetic contrast along the nano-rod axis in arbitrary units. Dotted lines indicate the position of the shadow. (**e**) Schematic of the T-XPEEM measurement (f) XMCD contrast profiles along the dotted line depicted in the XMCD images.

**Figure 6:**
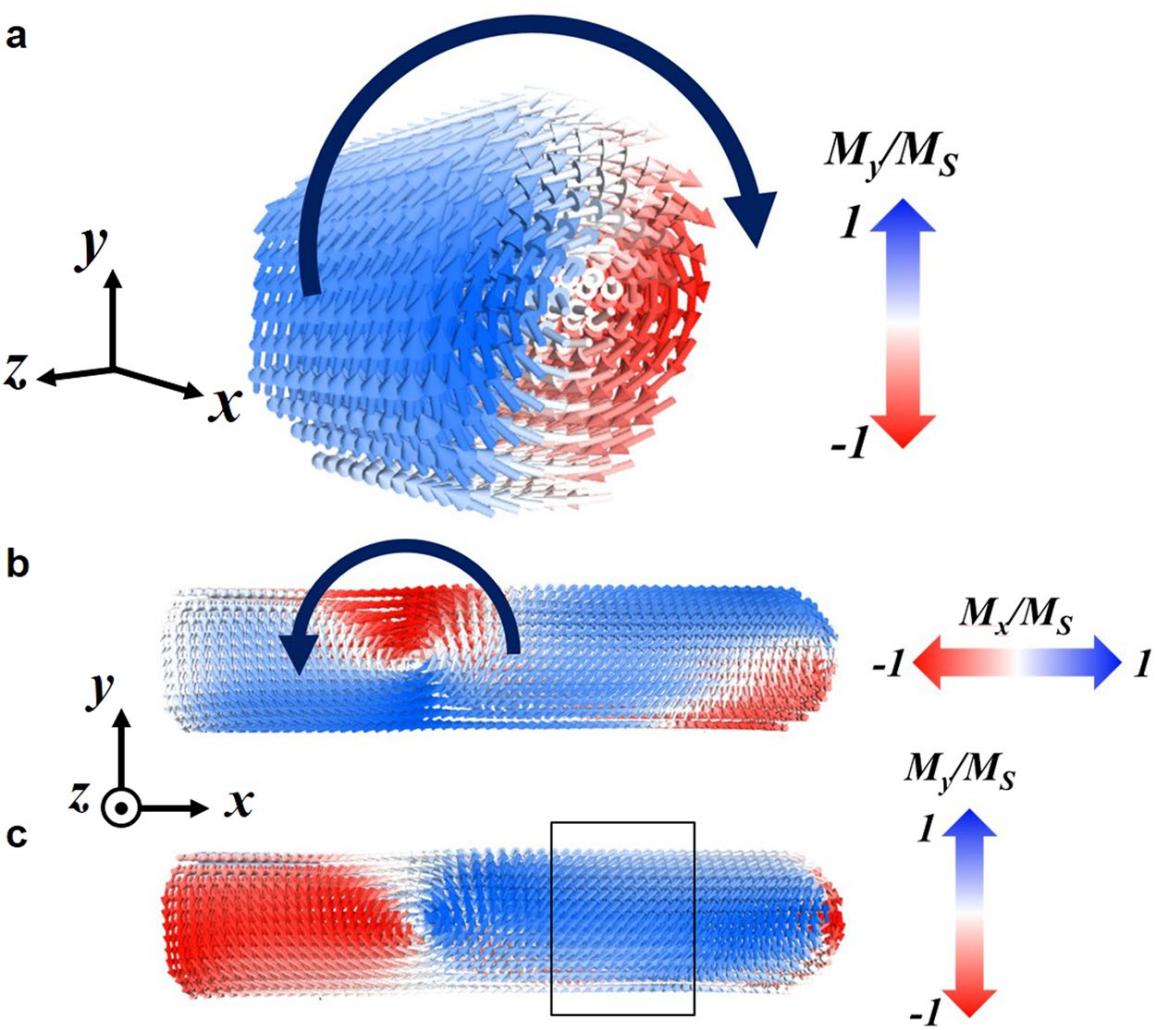
Micromagnetic simulations. 3D representation of the simulated remanent magnetic state for a Co nano-rod. (**a**) Magnetization map for a cross-section (rectangle in (**c**)). (**b**) and (**c**) Magnetization map using the magnetic component along (*M*_*x*_) and perpendicular (*M*_*y*_) to the nano-rod axis, respectively.

Individual magnetization structure of a Co nano-rod, piercing the SC film, has been visualized by transmission X-ray photoelectron emission microscopy (T-XPEEM), using X-ray magnetic circular dichroism (XMCD) as a magnetic contrast mechanism. Figure 5a–d shows the in-plane magnetic domain configuration of a single nano-rod, imaged by shadow contrast T-XPEEM, at different conditions. The geometry of the experiment (radiation impinging the sample at 16° grazing incidence) leads to the formation of a shadow close to the Co rods (Fig. 5e) that can be imaged and analysed by T-XPEEM^50–52^. The intensity at the shadowed areas depends on the absorption of the radiation at the bulk of the rods. Hence, the XMCD contrast appearing at the shadow is representative of the magnetic domain configuration of the bulk of the rod. Figure 5a shows the magnetic contrast obtained at 300 K, in remanence after a maximum applied field of −100 mT. A complex magnetic structure, with different magnetic domains is observed. Micromagnetic simulations have been used to elucidate the origin of this magnetic configuration. Figure 6 shows the simulated remanent state for a Co nano-rod obtained after applying a magnetic field of −100 mT parallel to the nano-rod axis (see the section methods for details). A complex 3D spin structure is found, in agreement with the magnetic contrast observed experimentally. Different magnetic domains are formed as an attempt to reduce the magnetostatic energy, mostly at the extremes of the nano-rod. The formation of this singular 3D magnetic state is induced by the polycrystalline nature of Co nanostructures grown by FEBID^42^. Two stable magnetic vortex states are formed, one with the core aligned parallel and the other perpendicular to the nano-rod axis (indicated by arrows in Fig. 6a and b respectively). The formation of the perpendicular vortex state is favored by the use of a saturation magnetization, *M*_*S*_, value similar to the pure Co. A reduction of *M*_*S*_, which is typically found in FEBID nanostructures with low Co content^42, 53^, induces a reduction of the exchange energy and the effect of the magnetostatic energy in the final magnetization configuration is more important. However, we found that even reducing the value of *M*_*S*_ to the half of the pure Co a vortex like state parallel to the longest dimension of the nano-rod is still present (see Supplementary Fig. S3).

To determine the effect of SC trapped magnetic fields, associated to supercurrent loops, on the stability of magnetic vortex states we compare the remanent magnetization structure of a Co nano-rod piercing the SC film above and below T_c_. Figure 5b shows the remanent magnetic state obtained after applying a maximum field of −50 mT at 300 K and Fig. 5c,d at 65 K. In (c) the field was applied at a high temperature and removed above *T*_*c*_ (Zero field cool, 65 K-ZFC) whereas in (d) it was applied below *T*_*c*_ (Field cool, 65 K-FC). Very similar contrast patterns were obtained in (a), (b), (c) indicating that the nano-rod remanent state does not significantly depend on the temperature, within the range evaluated, as expected considering the Co curie temperature. A different situation occurs when the nano-rod is magnetized at 65 K-FC, where the contribution of the SC trapped magnetic fields has to be considered in the remanent state. In this case (Fig. 5d) the magnetic contrast of the nano-rod is highly reduced. The contrast variation can be better observed by comparing the XMCD profiles shown in Fig. 5f. The obtained reduction of the magnetic signal along the nano-rod in 5 (d) would be consistent with a magnetic structure of a pure vortex state oriented perpendicular to the rod axis^52^. Micromagnetic simulations indicate that the parallel remanent vortex state disappear with applied magnetic fields of several mT. With this we demonstrate that the 3D nanometric magnetic structure of the Co nano-rods can be modulated by the SC stray fields. Thus, a proper choice of the SC sample geometry, and the associated current loops, could eventually lead to controllable 3D magnetic textures and vortex states at the nanometric scale.

In conclusion we have fabricated and analysed a novel hybrid system, based on a patterned high-pinning, high-temperature superconductor, combined with 3D ferromagnetic nano-rods. Our results demonstrate that such SC-FM hybrids offer the possibility to study mutual interaction between the stray magnetic fields generated due to the presence of SC current loops and the ones associated to the FM magnetization of nano-rods. We show that both the superconducting properties of the SC film and magnetic state of FM nano-rods can be tuned by cooperative non homogenous SC-FM stray fields. The results paved the way toward the ad-hoc design of hybrid systems in which the two stray field competition can yield new functionalities and phenomena such as the possibility of tailoring pinning of vortices in the SC or 3D magnetic domains in the nano-rods.

## Methods

High-quality YBa_2_Cu_3_O_7−d_ films were grown on LaAlO_3_ single crystal substrate by chemical solution deposition^41^. Transport bridges of 100 µm-length x 40 µm-width were patterned on the YBCO layer by optical lithography with the standard 4-point geometry. Nanometric antidots were patterned by Ga^+^ focused ion beam (FIB) lithography using 30 kV and 28 pA of ion beam voltage and current, respectively. Co rods were grown by focused electron beam induced deposition (FEBID) using 5 kV and 1.4 nA of electron beam voltage and current, respectively^43, 49, 54^. This technique allowed us to deposit locally cobalt nanostructures inside the antidots without damaging the surrounding YBCO matrix. Antidots and Co rods were patterned using a FEI Helios Nanolab 600 dual beam instrument FIB. This instrument is equipped with a field emission gun located along the vertical axis. The Ga^+^ focused ion beam column is located at 52° with respect to the electron column, and an individual gas injection system for the Co_2_(CO)_8_ precursor gas. To avoid residual Co impurities on top of the YBCO film, a soft Ar^+^ milling process (under 250 V) was carried out in a Sistec equipment using an ion dose of 0.5 mC/cm^2^, after the growth of the cobalt rods.

Electrical transport measurements were conducted in a Quantum Design 9 T PPMS cryostat with the applied magnetic field, *H*_*a*_, perpendicular to the film plane. The irreversibility line, *T*_*irr*_(*H*_*a*_), was determined from resistivity versus temperature curves, *ρ*(*T*), at different *H*_*a*_ by using a criterion of *ρ*(*T*_*irr*_)/*ρ*(95 K) = 10^−3^. For the critical current density determination, *J*_*c*_, we used a criterion of 100 µV/cm.

Numerical simulations based on the Magnetic Energy Minimization model in the critical state^55^, adapted for the thin film planar geometry^56, 57^ were used to determine the magnetic field distribution in the patterned YBCO bridge when subjected to an external magnetic field and applied electrical current. Static micromagnetic simulations to determine the magnetic distribution for an individual Co nano-rod were performed using the OOMMF code^58^. As magnetic parameters we used those that correspond to pure Co: saturation magnetization, *μ*_*0*_ *M*_*S*_ = 1400 kA/m; exchange constant, *A* = 30 × 10^−12^ J/m. The anisotropy constant was neglected (*K* = 0) because FEBID technique produces polycrystalline Co nanostructures composed by nanocrystals randomly oriented^59^, so the magneto-crystalline anisotropy does not contribute to the final state of the nano-rod. For these micromagnetic simulations, the three-dimensional shape of a Co nano-rod with length 888 nm long and circular cross-section of 200 nm diameter was built by stacking cubic cells of 5 nm lateral side (magnetic unit cell size). The simulated Co nano-rod has a rounded end with a curvature radius of 100 nm. To simulate the remanent magnetic state, the nano-rod is set in an initial magnetization state where all spins are perfectly aligned along the longest dimension of the rod and the simulation then runs by using a second order predictor corrector until the total energy of the system is minimized.

Magnetic domain imaging of cobalt nano-rods was done using of shadow contrast analysis in transmission X-ray magnetic circular dichroism (XMCD) and photo-electron emission microscopy (T-XPEEM)^50^ at the UE-49 PGMa beam-line (Synchrotron BESSY II). Magnetic contrast images were acquired at the Co L_3_-edge (707.6 eV), for different fields and temperatures. Measurements were performed with the magnetic field applied along the rod axis.

### Data availability

The data that support the findings of this study is available from the corresponding author upon request.

## Electronic supplementary material

Supplementary Information
