# Supplementary material for: Competition between Superconductor – Ferromagnetic stray magnetic fields in YBa2Cu3O7−x films pierced with Co nano-rods
Source: Sci Rep. 2017 Jul 18;7:5663. doi: 10.1038/s41598-017-05909-6 (PMC5516025; doi:10.1038/s41598-017-05909-6)
Supplement: Supplementary file 1 — Supplementary Information [file 41598_2017_5909_MOESM1_ESM.pdf]

Competition between Superconductor – Ferromagnetic stray magnetic fields in $YBa_2Cu_3O_{7-x}$ films pierced with Co nano-rods

V. Rouco[1], R. Córdoba[2], J.M. De Teresa[2,3], L. A. Rodriguez[4,8], C. Navau[5], N. Del-Valle[5], G. Via[5,9], A. Sánchez[5], C. Monton[6], F. Kronast[7], X. Obradors[1] and T. Puig[1], and A. Palau[1*]

Supplementary Information

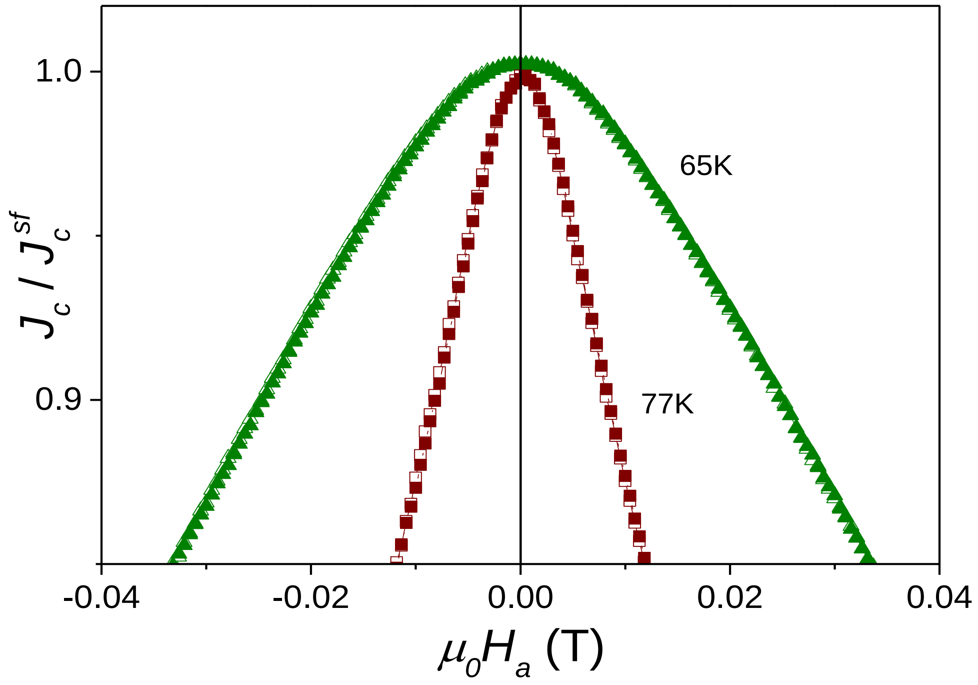

Figure S1. Non hysteretic J_c behaviour. Normalized critical current density vs. applied magnetic field at different temperatures for a pristine bridge measured by decreasing the magnetic field from 0.1T to -0.1T (closed symbols) and increasing it back to 0.1T (open symbols).

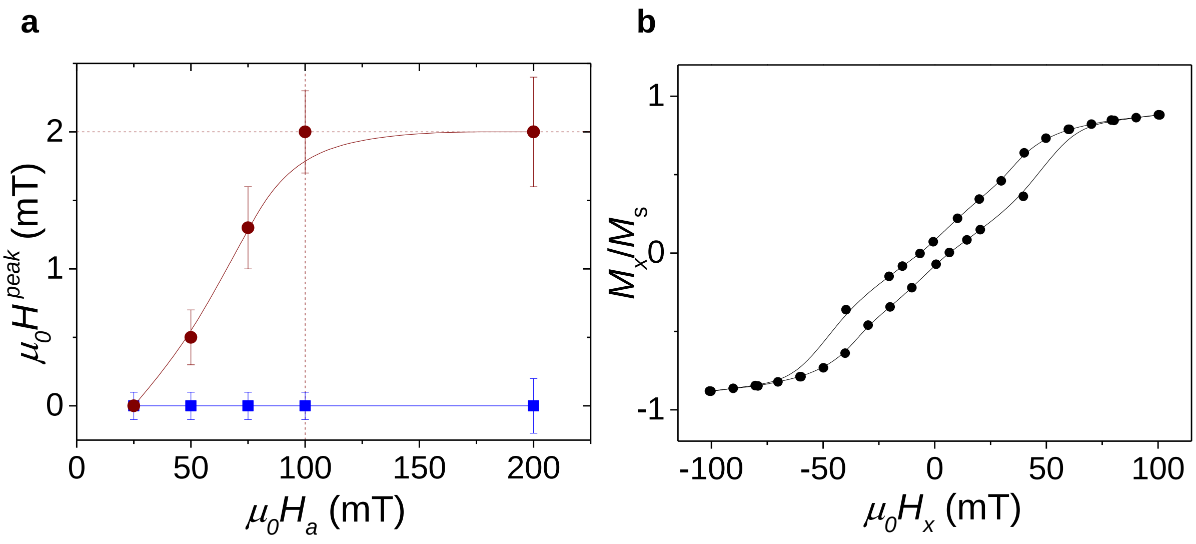

Figure S2. Magnetic behaviour of Co nano-rods. (a) Evolution of H^{peak}, evaluated from the position of maximum T_c in the irreversibility line, for different maximum applied fields obtained for a bridge with antidots (squares) and antidots filled with nano-rods (circles). Magnetic hysteresis loop of Co nano-rods obtained by micromagnetic simulations at 0K.

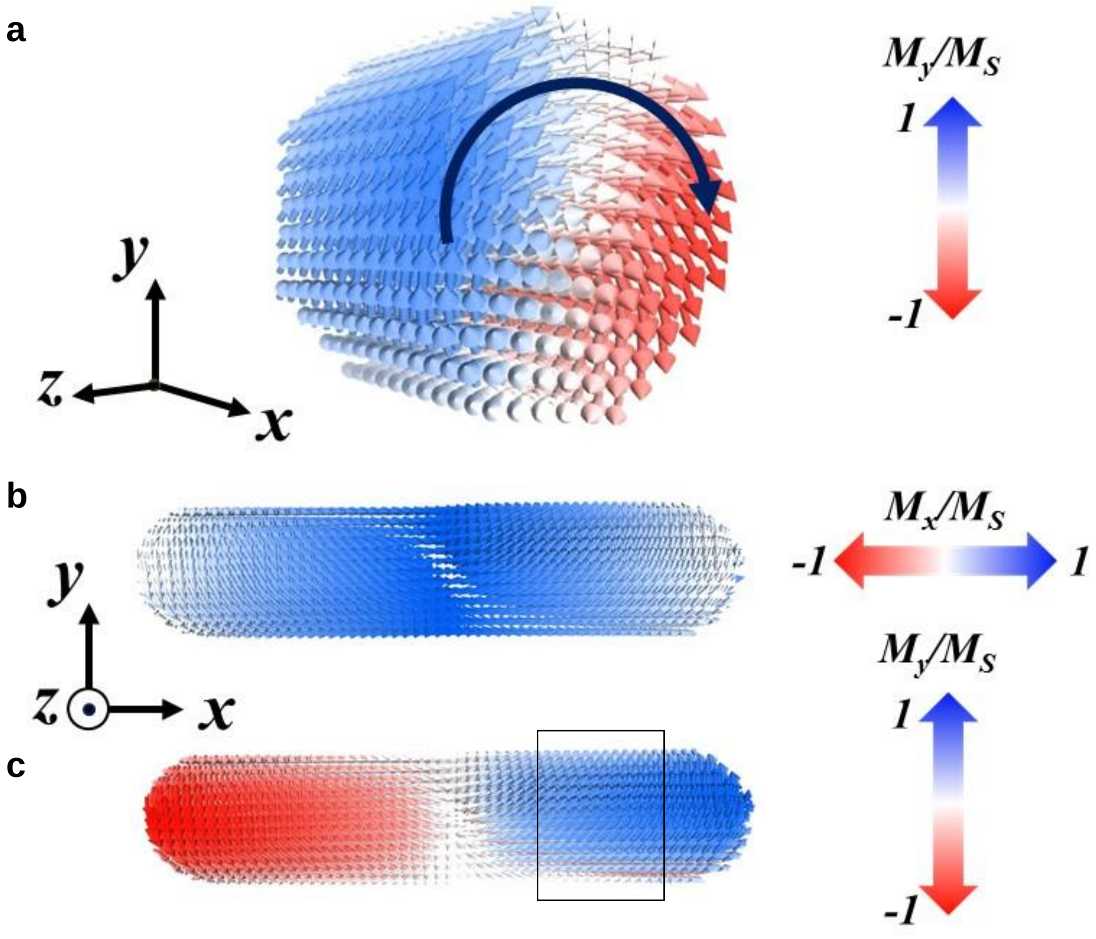

Figure S3. Micromagnetic simulations using a reduced M_S. 3D representation of the simulated remanent magnetic state for a Co nano-rod. (a) Magnetization map for a cross-section (rectangle in (c)). (b) and (c) Magnetization map using the magnetic component along (M_x) and perpendicular (M_y) to the nano-rod axis, respectively.
